# *Wolbachia* Infection Modifies the Profile, Shuttling and Structure of MicroRNAs in a Mosquito Cell Line

**DOI:** 10.1371/journal.pone.0096107

**Published:** 2014-04-23

**Authors:** Jaime G. Mayoral, Kayvan Etebari, Mazhar Hussain, Alexander A. Khromykh, Sassan Asgari

**Affiliations:** 1 Australian Infectious Disease Research Centre, School of Biological Sciences, The University of Queensland, Brisbane, Queensland, Australia; 2 Australian Infectious Disease Research Centre, School of Chemistry and Molecular Biosciences, The University of Queensland, Brisbane, Queensland, Australia; Natural Resources Canada, Canada

## Abstract

MicroRNAs (miRNAs) are small non-coding RNAs that play important roles in many biological processes such as development, cell signaling and immune response. Small RNA deep sequencing technology provided an opportunity for a thorough survey of the miRNA profile of a mosquito cell line from *Aedes aegypti*. We characterized the miRNA composition of the nucleus and the cytoplasm of uninfected cells and compared it with the one of cells infected with the endosymbiotic bacterium *Wolbachia* strain *w*MelPop-CLA. We found an overall increase of small RNAs between 18 and 28 nucleotides in both cellular compartments in *Wolbachia*-infected cells and identified specific miRNAs induced and/or suppressed by the *Wolbachia* infection. We discuss the mechanisms that the cell may use to shuttle miRNAs between the cytoplasm and the nucleus. In addition, we identified piRNAs that changed their abundance in response to *Wolbachia* infection. The miRNAs and piRNAs identified in this study provide promising leads for investigations into the host-endosymbiont interactions and for better understanding of how *Wolbachia* manipulates the host miRNA machinery in order to facilitate its persistent replication in infected cells.

## Introduction

MicroRNAs (miRNAs) are small non-coding RNAs of ∼22 nucleotides (nt) produced by animals and plants as well as some viruses. miRNAs may be encoded from non-coding transcripts, introns or even coding regions. miRNA genes are expressed mainly by RNA polymerase II in the nucleus as primary miRNA (pri-miRNA) [1], [2]. pri-miRNA is cleaved by Drosha to a 60- to 80-nt precursor miRNA (pre-miRNA) with multiple mismatches and bulges, which is then exported by Exportin-5 to the cytoplasm and cleaved by Dicer-1, generating an approximately 22-nt double-stranded miRNA (miRNA-5p: miRNA-3p duplex). After the sequential processing of miRNA precursors, mature miRNA joins specific Argonaute proteins (AGO) in what is referred to as an miRNA-induced silencing complex (miRISC) and guides miRISC to complementary mRNA sequences to regulate their expression [1], [3], [4]. In plants, miRNAs usually pair nearly perfectly with target sites, while in animals complementarity of miRNAs with their target sequences is partial [4]. Perfect pairing between a target and nucleotides 2–8 from the 5′ end of the miRNA (seed region) usually plays a significant role in target recognition [3], [5].

The main function of miRNAs is the regulation of gene expression at the post-transcriptional level. miRNAs repress or up-regulate most of the genes that they control by a small amount. Yet, collectively they affect nearly all the cellular pathways, from development to oncogenesis [1]. In the last few years, investigators have studied the involvement of miRNAs in host-pathogen interactions. This involvement is now well established, but the amount of studies in insects is limited. Their role has been shown by disrupting the biosynthesis of host miRNAs, inhibiting specific miRNAs or by studying the miRNA profile of the host before and after infection [6]. These changes in the miRNA levels may be induced by pathogen-derived components (including their own miRNAs) or as an immune response mechanism to infection [1].

*Wolbachia* are gram-negative endosymbiotic bacteria that are transmitted maternally and potentially infect more than 40% of all insects [7]. The bacteria’s impacts on the host are variable, but they are best known for i) the manipulation of the host reproductive system; however, in some cases this relation has evolved to symbiotic rather than parasitic [8]; ii) inhibition of replication of several vector-borne pathogens including arboviruses such as dengue and chikungunya viruses in *Aedes aegypti* by some strains of this bacterium, and the human malaria parasite *Plasmodium falciparum* after the successful establishment of *Wolbachia* in an important malaria vector, *Anopheles stephensi* [9]. This places *Wolbachia* as potential agent for the control of insect-transmitted diseases. Despite the relevance of these discoveries, the molecular mechanisms behind these effects are largely unknown and need to be elucidated.

There are currently only three studies that have elucidated the involvement of miRNA in the interaction between *Wolbachia* and its host, *Ae. aegypti*. Using microarrays, Hussain et al. (2011) found that 35 miRNAs changed their expression levels between tetracycline-treated (Tet) and *w*MelPop-CLA infected (Pop) mosquitoes. They showed how *Wolbachia* up-regulates the abundance of aae-miR-2940 and in turn it up-regulates the transcript levels of the host *metalloprotease f41 ftsh* to guarantee its persistence within the host cells [10]. A subsequent study showed that infection with *Wolbachia* supresses the expression of the *AaDnmt2 DNA methyltransferase* gene involved in host defence and gene regulation and that infection with dengue virus (DENV) induces it [11]. Interestingly, aae-miR-2940 down-regulates the expression of *AaDnmt2*. A reduction of the transcript levels of *AaDnmt2* by the miRNA caused an inhibition of *Wolbachia* replication, but promoted the replication of DENV. Experimental results suggested a causal link between the *Wolbachia* manipulation of aae-miR-2940 and the blocking of DENV replication in *Wolbachia* infected cells. Finally, Osei-Amo et al (2012) found that the up-regulation of aae-miR-12 in infected cells with *Wolbachia* affects the transcript levels of *DNA replication licensing* (*MCM6*) and *monocarboxylate transporter* (*MCT1*) genes. Using a specific inhibitor (antagomirs) against aae-miR-12, *Wolbachia*’s dependence on this miRNA to guarantee its persistence within the host cell was demonstrated [12].

To further investigate the manipulation of host mosquito miRNAs by *Wolbachia*, in the present work, we provide a comprehensive analysis of the miRNA profile in the nucleus and the cytoplasm of *Ae. aegypti* Aag2 cells and how the infection with *Wolbachia* alters this composition. We study how *Wolbachia* infection changes the levels of some of the most abundant miRNAs and identify key miRNAs that are highly regulated and could play important roles in immunity and defense in mosquito cells.

## Materials and Methods

### Insect Cell Lines Culture

*Ae. aegypti* Aag2 cells were maintained in growth media in a 1∶1 mixture of Mitsuhashi–Maramorosch and Schneider’s insect media (Invitrogen) supplemented with 10% FBS. Aag2 cells infected with the *w*MelPop-CLA strain of *Wolbachia* (+Wol) were maintained as Aag2 cells. +Wol cells have been maintained as infected for the last three years in the lab with regular replenishing of the culture from liquid nitrogen stocks. *Wolbachia* density was regularly examined by qPCR using specific primers to the *wsp* gene (wsp-F, 5′- ATCTTTTATAGCTGGTGGTGGT-3′; wsp-R, 5′-GGAGTGATAGGCATATCTTCAAT-3′).

### Nuclear and Cytoplasmic RNA Extractions and Small RNA Sequencing

Cytoplasmic and nuclear fractions were isolated using PARIS kit (Ambion) according to the manufacturer’s instructions. Briefly, cells were washed with PBS, pelleted at low speed and gently resuspended in 300 µl of ice-cold cell fractionation buffer, incubated on ice for 10 min, centrifuged at 2000 rpm at 4°C for 5 min. The pellet contained the nuclear fraction that was resuspended in 300 µl cell disruption buffer and vortexed vigorously. The supernatant contained the cytoplasmic fraction. RNA from both nuclear and cytoplasmic fractions was extracted with 2× lysis/binding buffer followed by using filter cartridges and eluted in 50 µl heated elution buffer supplied in the kit. RNA concentrations were measured using a spectrophotometer, and integrity and separation of fractions was ensured through analysis of RNA on a 1% (w/v) agarose gel (Figure S1).

A small RNA library was generated from all samples using the Illumina Truseq Small RNA Preparation kit at LC Sciences (Houston, USA). The purified cDNA libraries were sequenced on Illumina GAIIx and raw sequencing reads (36 nts) were obtained using Illumina’s Sequencing Control Studio software version 2.8 followed by real-time sequencing image analysis and base-calling by Illumina’s Real-Time Analysis version 1.8.70. Deep sequencing data have been deposited in NCBI’s Gene Expression Omnibus [13] and are accessible through GEO Series accession number GSE55210.

### Computational Pipeline for Predicting miRNAs

In order to filter data from impurity sequences, reads without 3′ adaptors and also reads with less than 16 nt were discarded and clean data considered as mappable reads for further analysis. Tab separated files with the read sequences and their counts were used as input file for further analysis with miRanalyzer [14]. An updated version of miRanalyzer, a web-based server for the detection of known and prediction of novel miRNAs was used for this analysis. This software is based on a random forest classifier and implements a highly accurate machine learning algorithm (Support Vector Machine) to predict new miRNA candidates from high throughput sequencing data [15]. In brief, to analyze differential expression of miRNAs in two libraries, miRanalyzer uses an R package (DESeq) to count read data. First, each library is analyzed individually and then the software combines the reads to reach a normalization value for each dataset. After this stage, a new Normalized Expression Value is defined for each miRNA. Due to software limitation, a maximum of two groups can be compared at the same time and the normalized value can be different for each miRNA when one of those libraries would be compared with another dataset.

The ultrafast short read aligner Bowtie was used to align the reads to the *Ae. aegypti* genome and miRNA database. We allowed a maximum of two mismatches in the genome, and known and homologous miRNA databases in our mapping parameters. Also, one mismatch was implemented for other transcribed libraries such as Rfam and Repbase. The software’s default seed alignment length for Bowtie (17 for known miRNA, 19 for genomes and 20 for other transcribed libraries) was selected for all the analyses. Reads mapped to the RepBase (www.girinst.org/repbase) and Rfam (http://rfam.sanger.ac.uk) were removed before further analysis. To find conserved miRNAs, all short reads from deep sequencing were mapped to *Ae. aegypti* known miRNA sequences as the primary source of reference. We previously described the approach for the identification of novel miRNAs and the criteria to predict their pre-miRNA structures from genome sequences [16].

Differential expression of miRNAs between two conditions was analysed based on the DESeq package [17] on miRanalyzer server. Normalization of sequence counts for each sample was achieved by dividing the counts by a library size parameter of the corresponding library and the final fold change values were given in log2 scale.

### RNA Extraction, cDNA Synthesis and qPCR

Total RNA from Aag2 and Pop cells was isolated using Tri-Reagent (Ambion Inc.), and subsequently treated with DNase I before being used for reverse transcription. A total of 2 µg of RNA for each sample was reverse transcribed using oligo dT in a total volume of 20 µL. qPCR with AGO1 (forward: 5′-CGCAGACAAGAAGGAACAGA-3′ and reverse: 5′-TCCCACAGGACGTGATAATG-3′), AGO2 (forward: 5′-CTGGACATGACTTGCCTGAA-3′ and reverse: 5′-AGCTCATGGTTGCTTCCAAT-3′) and AGO3 (forward: 5′-GTTATAATCTGCCCAACGTC-3′ and reverse: 5′-CCCAGTTTGCAGTTCATCTG-3′) gene-specific primers was performed to determine their mRNA levels in Aag2 and Pop cells. Platinum SYBR Green Mix (Invitrogen) with 1 µL of the first-strand cDNA reaction was used in a Rotor-Gene thermal cycler (Qiagen) under the following conditions: 95°C hold for 30 s, then 40 cycles of 95°C for 15 s, 50°C for 15 s, and 72°C for 20 s, followed by the melting curve (68–95°C). Three biological replicates with three technical replicates were analysed. The *RPS17* gene was used for normalization of the RNA templates. The Student t test was used to compare the differences in means.

### Northern Blotting of Small RNAs

Cytoplasmic and nuclear fractions were isolated from *w*MelPop-CLA infected and non-infected *Ae. aegypti* mosquitoes, and subsequently total RNA was extracted from the fractions as described above. The RNA samples were run on 15% urea denaturing polyacrylamide gel, electroblotted to a nylon membrane by a semi-dried Western blotting apparatus (Bio-Rad), and UV cross-linked. DNA oligonucleotides reverse complementary to specific miRNA sequences were labelled with [α-^32^P]-dCTP using terminal nucleotide transferase. All probe hybridizations and washings were done under stringent conditions at 50°C. Blots were exposed to a phosphorimager screen overnight, and radioactive signals were detected using a phosphorimager scanner (Storm). For removing the old probe, blots were washed in boiling 0.1% SDS twice for 15 min each time. Stripping of the probe was confirmed by scanning the blots as described above.

## Results and Discussion

### Deep Sequencing of Small RNAs

**Figure 1:**
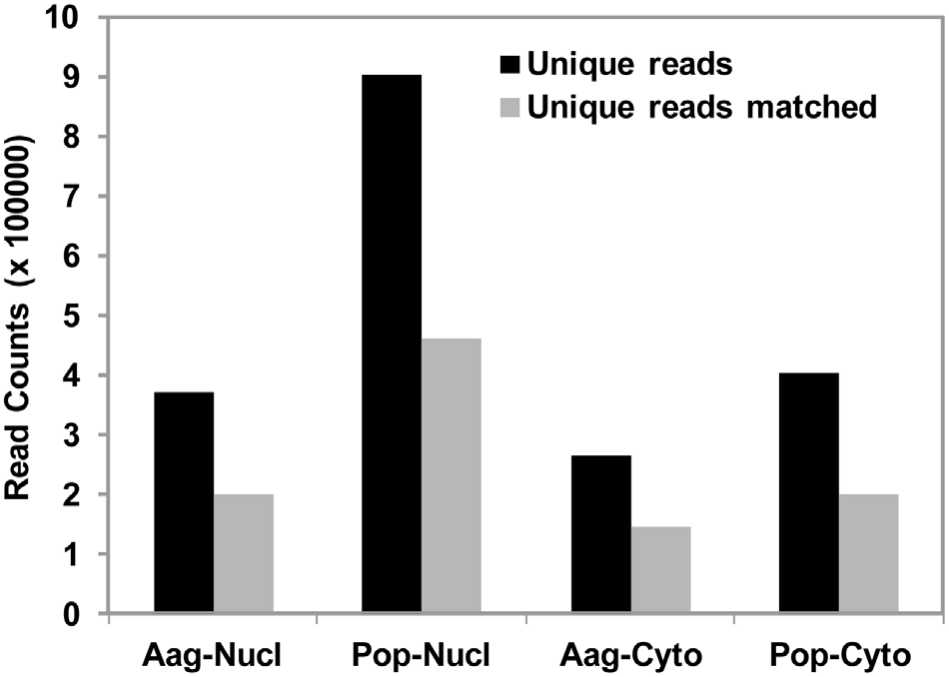
Number of unique reads and unique reads that matched the *Ae. aegypti* genome from the four libraries deep sequenced using RNA samples from the nucleus and the cytoplasm of Aag2 and Pop cells.

**Figure 2:**
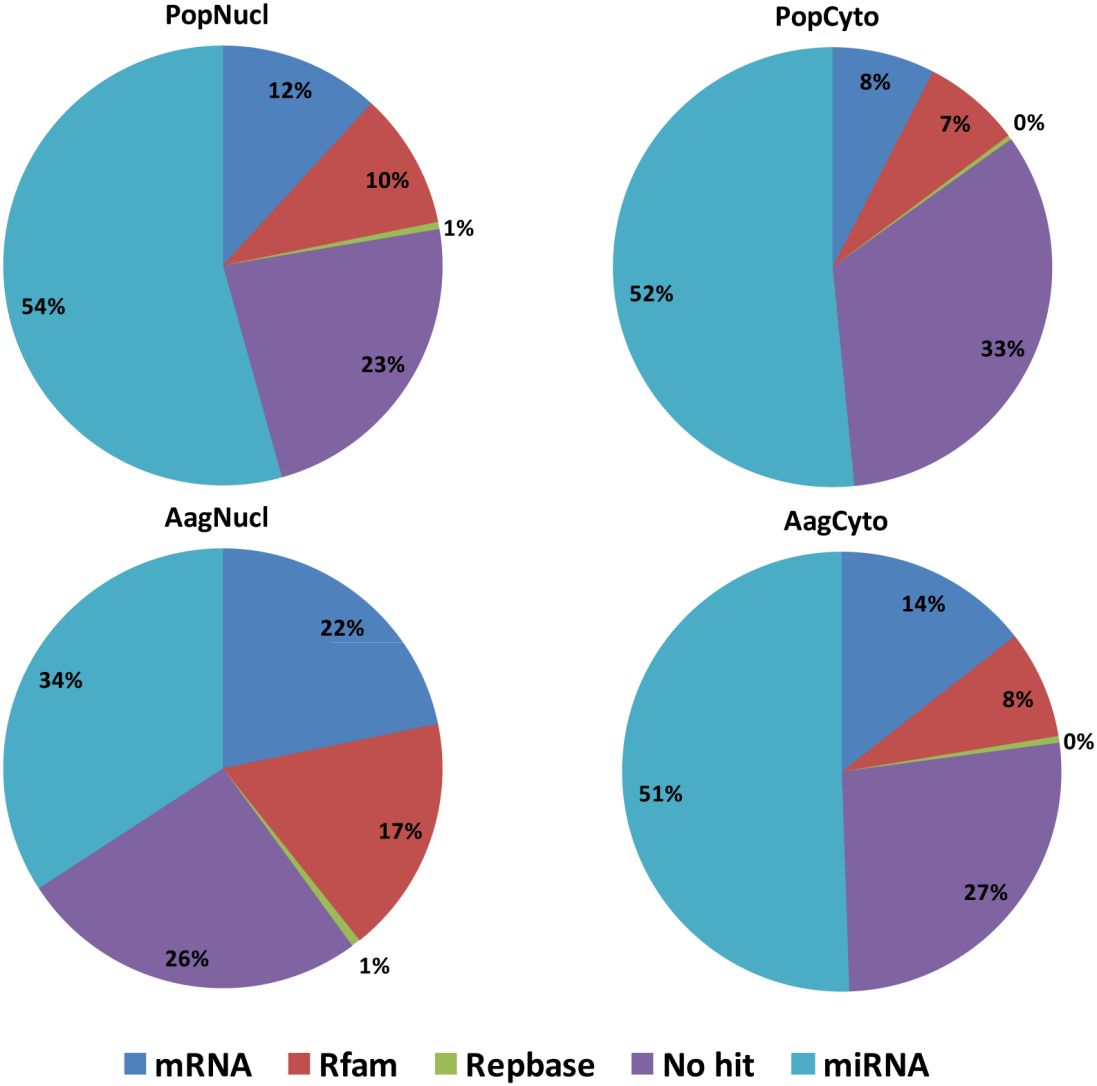
Small RNA deep sequencing reads statistics for the four libraries deep sequenced using RNA samples from the nucleus and the cytoplasm of Aag2 and Pop cells.

Illumina small RNA deep sequencing platform was used to characterize and compare the abundance of miRNAs in the nucleus and the cytoplasm of Aag2 cells with and without *Wolbachia* strain *w*MelPop-CLA (Pop) infection. We obtained 23,755,504 and 5,129,315 reads from the nuclear and cytoplasmic fractions of Aag2 cells, respectively and 28,255,577 and 9,069,354 reads for Pop cells in the same cellular compartments, respectively. We removed the reads with less than 15 bases, low quality reads and reads without a 3′ adaptor. Reads mapped to Rafm and Repbase (7–18%) were discarded before any further analysis. Figure 1 shows the number of reads for each library after the adaptor removal and also the number of reads that mapped to the *Ae. aegypti* genome. Approximately, 50% of reads were mapped to known miRNAs when they were blasted against miRBase v20; this percentage was considerably lower (34%) in the nuclear fraction of Aag2 cells (Figure 2). The percentage of reads that mapped to protein coding mRNA sequences was 8–22% of all clean reads for each library, which were considered RNA degradation products and were also discarded (Figure 2).

### Nucleus and Cytoplasm of Mosquito Cells have Different miRNA Profiles

**Figure 3:**
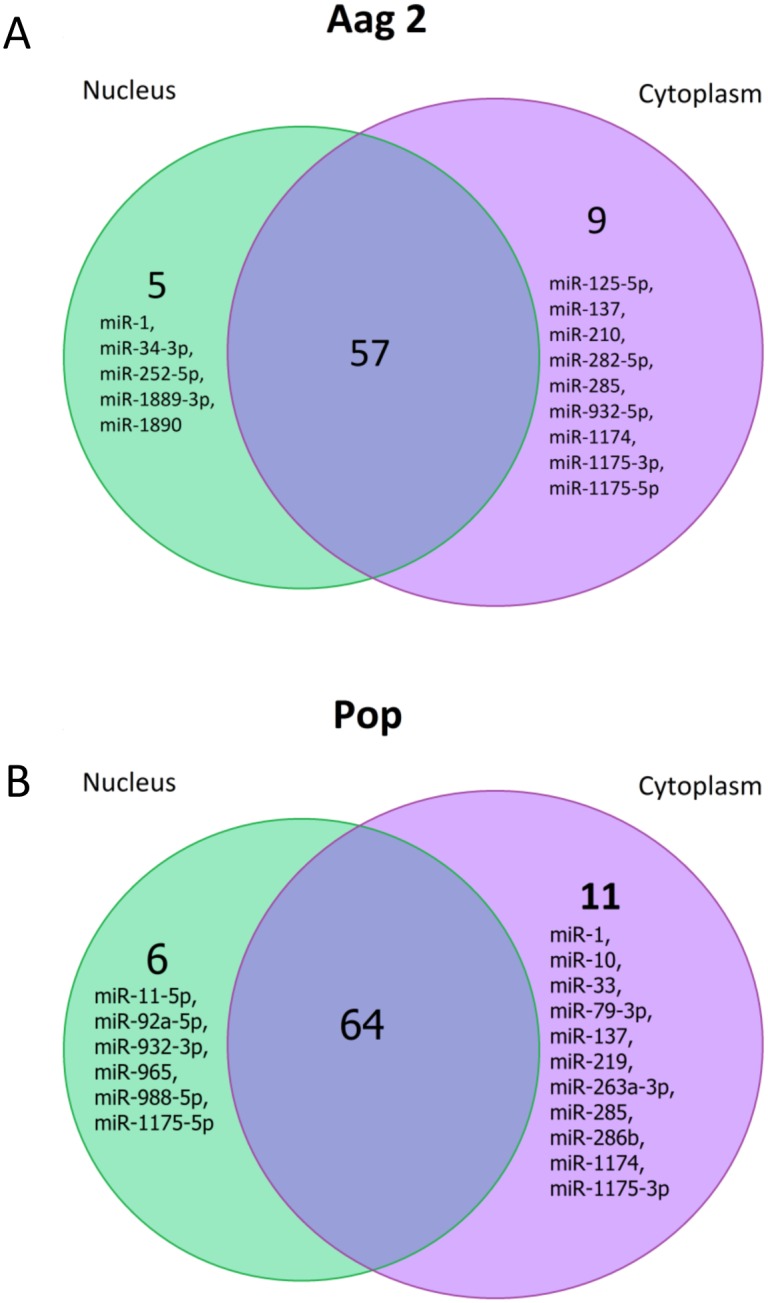
Differentially expressed miRNAs in the nucleus and the cytoplasm of (A) Aag2 cells and (B) cells infected with *Wolbachia* (Pop).

**Figure 4:**
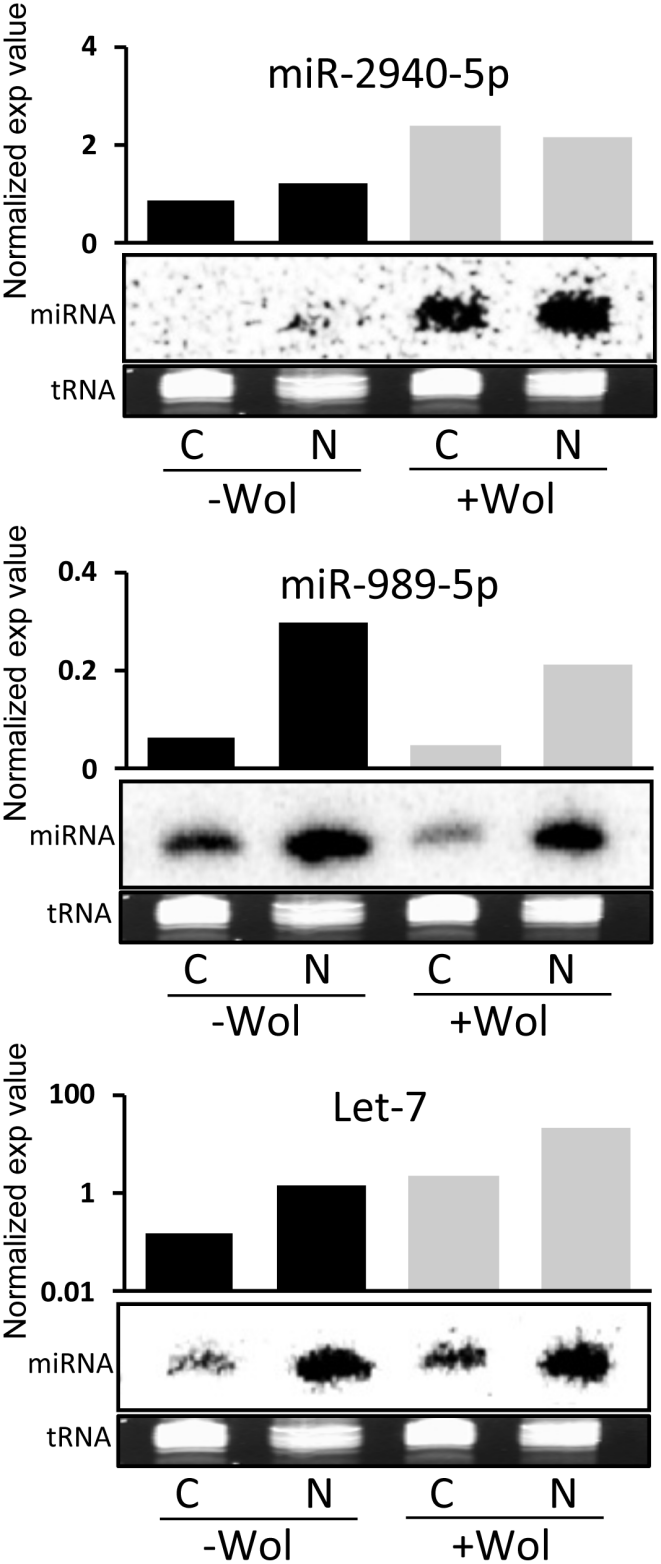
Verification of deep sequencing data. Northern blot analysis of RNA extracted from nuclear and cytoplasmic fractions of *w*MelPop-CLA infected (+Wol) and non-infected (–Wol) *Ae. aegypti* whole mosquitoes and their relevant read counts from the small RNA deep sequencing. The same blot was used multiple times after removal of probe. tRNA is shown as loading control. C, cytoplasmic fraction; N, nuclear fraction.

Globally, 71 different miRNAs in Aag2 cells were identified; 62 were detected in the nucleus and 66 in the cytoplasm (Table S1). Fifty seven miRNAs were found in both compartments, but miR-1, miR-34-3p, miR-252-5p, miR-1889-3p and miR-1890 were detected only in the nucleus; nine other miRNAs were detected solely in the cytoplasm; miR-125-5p, miR-137, miR-210, miR-282-5p, miR-285, miR-932-5p, miR-1174, miR-1175-3p and miR-1175-5p (Figure 3A). In the data obtained from cells infected with *Wolbachia*, 81 different miRNAs were identified and 64 of those were detected in both cellular compartments. miR-11-5p, miR92a-5p, miR-932-3p, miR-965, miR-988-5p and miR1175-5p were expressed in the nucleus and 11 in the cytoplasm: miR-1, miR-10, miR-22, miR-79-3p, miR-137, miR-219, miR-263a-3p, miR-285, miR-286b, miR-1174 and miR-1175 (Figure 3B; Table S1). Our data suggested that the profiles of miRNAs in both compartments were quite different and that the presence of *Wolbachia* induced the synthesis of new miRNAs and therefore changes in the host miRNA composition. To verify the deep sequencing data, we analyzed the abundance of a number of randomly selected miRNAs in the nuclear and cytoplasmic fractions of *Wolbachia*-infected and non-infected whole mosquitoes by Northern blotting and found that the trend in changes in the abundance of the miRNAs were consistent between the blots and the expression values (Figure 4). However, miR-2940-5p, which was found in all the four libraries at various levels, was almost absent in RNA samples from mosquitoes without *Wolbachia* when analyzed on the Northern blot. Nevertheless, this is consistent with our previous observations that miR-2940-5p is exclusively found in *Wolbachia*-infected mosquitoes, but present in Aag2 and C6/36 non-infected cell lines, although at lower levels compared to their respective *Wolbachia*-infected cells [10], [11]. Numerous studies have shown that cell lines are different to whole organisms that consist of several tissues and cell types [18]–[21]. However, when homogenous infection of cells is considered, cell lines could provide useful information. To the best of our knowledge, this is the first comprehensive study on the expression and changes of miRNAs in the nucleus and the cytoplasm and how they change after being challenged with a pathogen.

Since the machinery necessary for the final steps of the processing and maturation of miRNA is located in the cytoplasm, finding nucleus-specific miRNAs may look contradictory from a biosynthetic point of view. However, as described above, we found several miRNAs solely in this compartment and similarly, we have identified others that are highly abundant in the nucleus but had a low copy number in the cytoplasm. A good example is miR-276-3p, which copy number in the nucleus and the cytoplasm were 11,287 and 1,197, respectively. The question arises as to how these mature miRNAs could be detected in high quantities in the nucleus (and at different levels and composition from those in the cytoplasm) and whether they are selectively transported to the nucleus. This will be discussed in more details in the section “miRNA trafficking between the nucleus and the cytoplasm”.

### *Wolbachia* Enhances the Biogenesis of Small RNAs and AGO mRNAs

**Figure 5:**
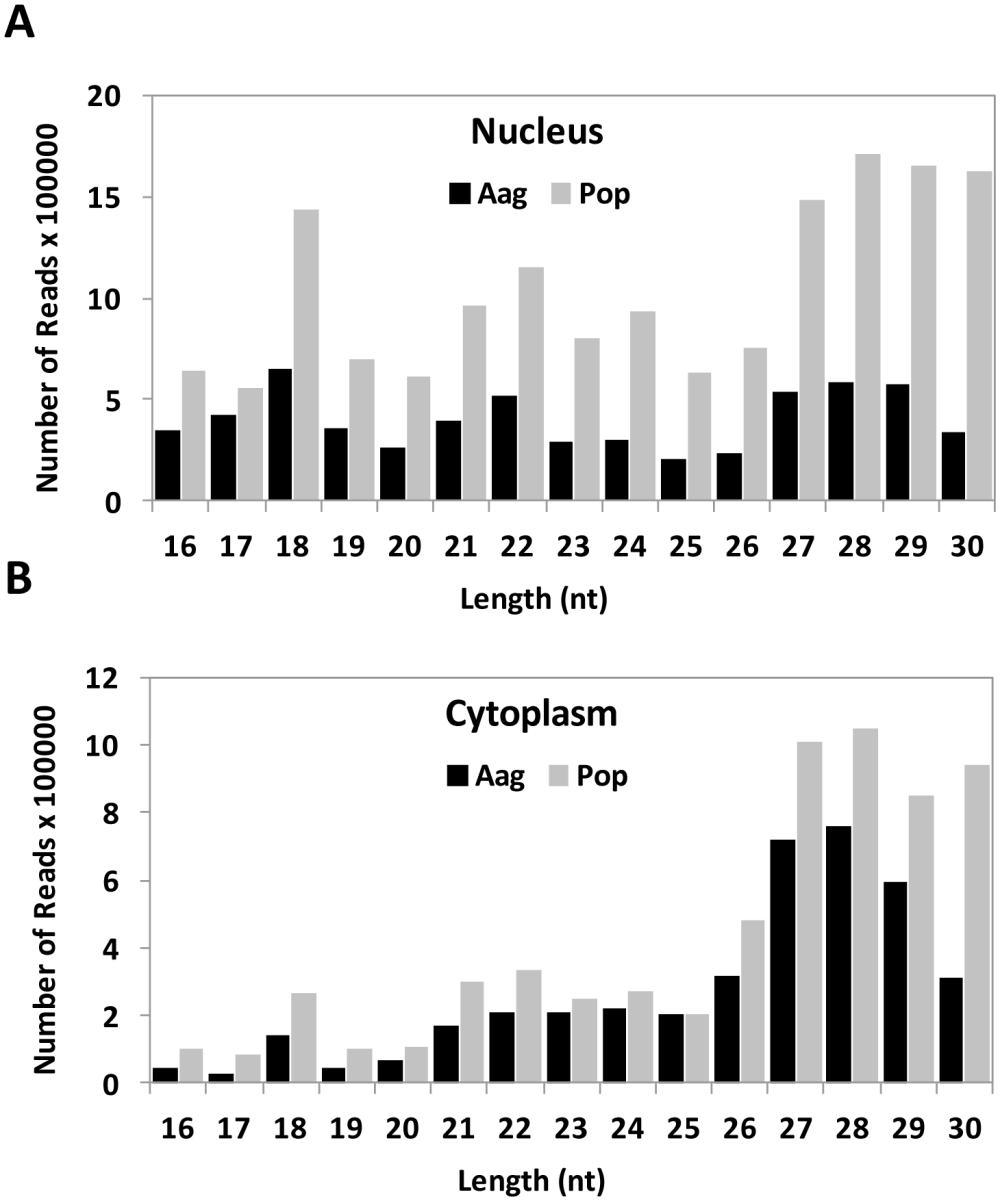
Length distribution of mappable reads obtained from deep sequencing from the four libraries deep sequenced using RNA samples from the nucleus (A) and the cytoplasm (B) of Aag2 and Pop cells.

The sequence length distribution of small RNAs (sRNAs) for the four deep sequenced libraries are shown in Figure 5A and 5B. In the nucleus of Aag2 and Pop libraries, there were two well-defined peaks at 18 and 22 nt, and some other smaller peaks flanking those lengths, which is the typical range for mature miRNAs. The two samples shared a similar pattern; however, in *Wolbachia*-infected cells there was an overall increase in the number of all small RNAs between 15 and 30 nt. This was also true for the data obtained in the cytoplasm (Figure 5B). In the cytoplasm, reads with 18–22 nt were less abundant than those with 26–30 nt, which correspond to the PIWI-Interacting-RNAs (piRNAs). piRNA-like sequences were more abundant than miRNA-like sequences regardless of the presence of *Wolbachia*. piRNA is a distinct class of small RNAs with a larger size than miRNA and short interfering RNA (siRNA). They do not originate from hairpin-shaped precursors (different from miRNA) or double stranded RNAs (difference with siRNA). piRNAs interact with PIWI proteins and produce piRNA-induced silencing complex (piRISC), which can bind to RNAs in animal germ lines [22]. These complexes have been shown to be associated with post-transcriptional gene silencing and epigenetic of retrotransposons in germline cells [23]. Traditionally, piRNAs were not associated with host-pathogen associations, but recent studies have shown that piRNAs could have an important role in this interaction [24]–[27]. Although the number of piRNA sequences were considerably higher in the cytoplasm in comparison to other sizes, there was a smaller peak at 18 nt and another at 21–22 nt, especially after *Wolbachia* infection (Figure 5B). In both cellular compartments, the sequence reads at 27–30 nt were clearly induced after infection.

**Figure 6:**
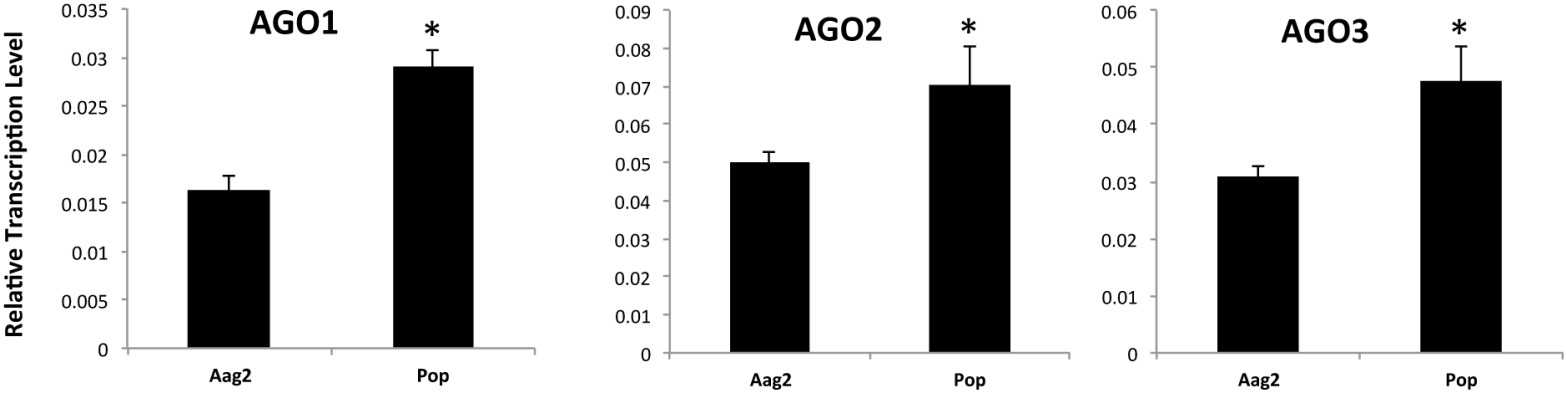
RT-qPCR analysis of mRNA transcript levels of Argonaute 1–3 (AGO1–3) in Aag2 and Pop cells. For each sample, three biological replicates with three technical replicates were analyzed (*p<0.05).

After the finding that *Wolbachia* induced the overall levels of sRNAs of most sizes, we hypothesized that the machinery involved in its processing and loading could be affected. Using RT-qPCR, we quantified the mRNA levels of the Argonaute proteins AGO1–3 in non-infected and infected cells with *Wolbachia*. Consistent with our deep sequencing data, there was an overall increase in the mRNA levels of the three AGOs (Figure 6). We have found only another study that studied the response of AGO proteins after the challenge with a pathogen. Wang et al. (2013) characterized the four members of the Argonaute family AGO1, AGO2, AGO3 and PIWI in *Bomyx mori*, and using microarray, they studied changes in these proteins in response to infection with the baculovirus *Bombyx mori* nucleopolyhedrovirus (BmNPV) or *Escherichia coli* [28]. Six hours after infection with BmNPV, the expression of AGO1–3 and PIWI were up-regulated 2.8 to 5.0 times, while *E. coli* infection induced 1.5-fold increase in the expression of both AGO2 and PIWI. It is likely that an increase in the levels of AGO proteins is a general response to infection. This increase in AGO proteins together with higher levels of available small RNAs (Figure 5A and 5B) may result in a more efficient defence response by enhancing immunity and activating the RNAi pathway. In addition, these data suggest that there may be differences in the type of AGO proteins that become involved depending on the nature of the infection.

### *Wolbachia* Changes the Host’s miRNA Profile

#### Most abundant miRNAs

**Figure 7:**
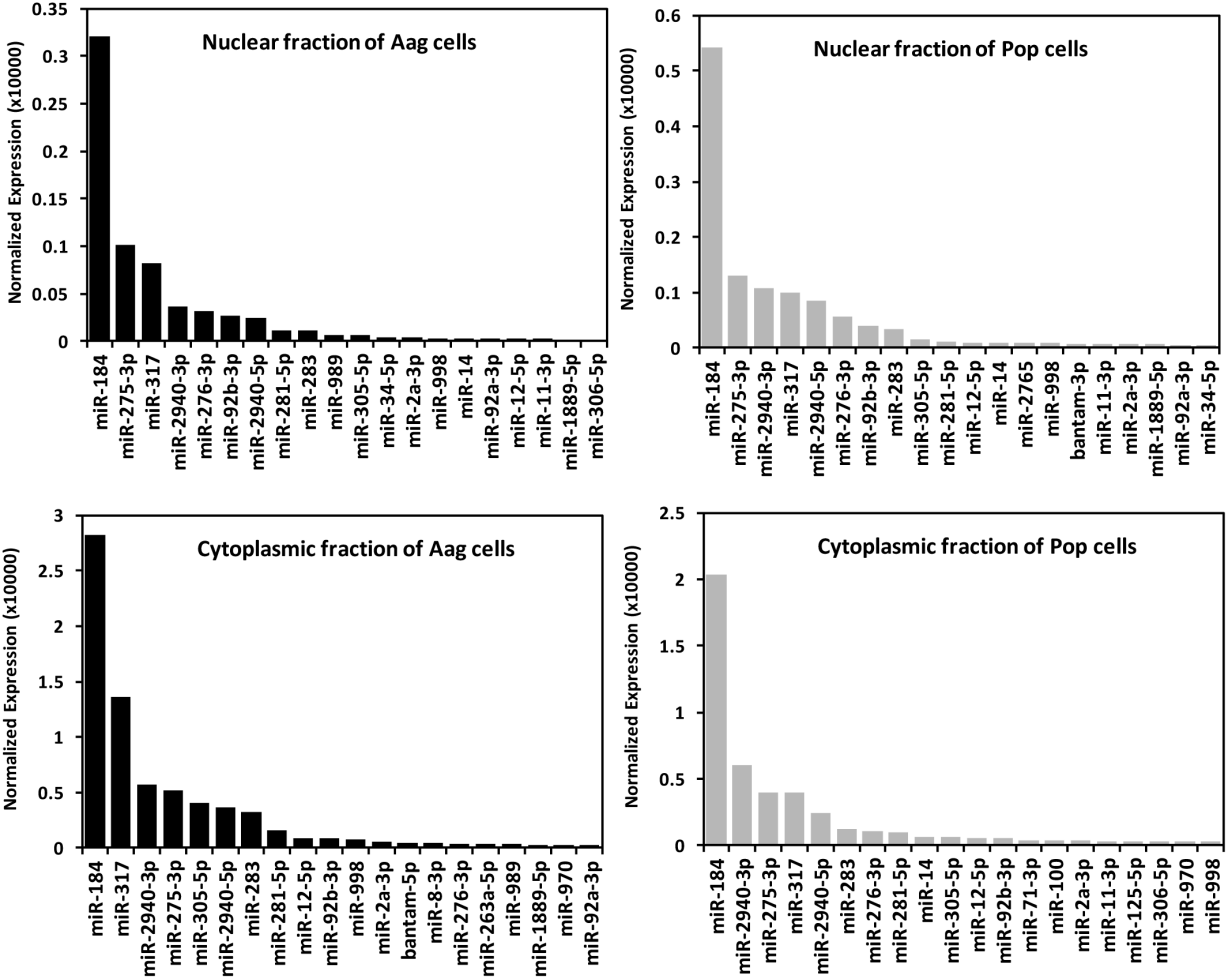
Read numbers of the twenty most abundant miRNAs from the four libraries deep sequenced.

The most abundant miRNAs in the nucleus and the cytoplasm of Aag2 are shown in Table 1. miR-184, miR-317, miR-2940-3p and miR-275 were the four most abundant miRNAs in the four libraries sequenced; however, while miR-317, miR-2940-3p and miR-275 changed their relative position among the different libraries, miR-184 was always the most abundant with a significant difference in the number of reads when compared to the second most abundant miRNA (Figure 7). miR-184 has been identified in over 39 organisms [29] and is conserved from humans to insects. It has been reported as one of the most abundant miRNAs in several orders of insects [29]–[31]. Studies in flies have shown that it may be involved in development, including tissue fate establishment, differentiation and maintenance of tissue identity [32]. miR-317 and miR-275 were reported among the most abundant miRNAs in *Culex quinquefasciatus* and *Aedes albopictus* [29]. This study together with our results suggest that miR-317 and miR-275 may be among the most abundant miRNAs in mosquitoes; other studies in Lepidoptera highlighted these miRNAs as being regulated upon infection/parasitization, but they were not among the most abundant miRNAs [31], [33]. No function for miR-317 is still known but in *Drosophila*, miR-275 was reported to be involved in the stem cell differentiation pathway through regulating *Bam*; this regulation is crucial for the proper spermatid terminal differentiation [34]. In mosquitoes, miR-275 was identified to play an essential role in diverse pathways such as blood digestion, fluid excretion and egg development [35]. It is remarkable that miR-2940 is present among the four most abundant miRNAs because, based on miRBase, it seems to be a mosquito specific miRNA. There are already two reports that studied and highlighted the relevance of this miRNA in the interaction of the host with *Wolbachia*. Hussain et al (2011) described how *Wolbachia* manipulates the levels of this miRNA in *Ae. aegypti* mosquitoes in order to guarantee its persistence and survival in the mosquito cells [10]. Also, the up-regulation of miR-2940 in *Wolbachia*-infected cells leads to down-regulation of the DNA methyltransferase 2 (*AaDnmt2*) transcript levels, and this results in a reduction in the replication of DENV and an increase in *Wolbachia* replication [11].

**Table 1:** Twenty most abundant miRNAs in the nucleus and the cytoplasm of Aag2 cells versus *Wolbachia*-infected cells (Pop).

	Nucleus		Cytoplasm	
	Aag2	Pop	Aag2	Pop
**1**	aae-miR-184	aae-miR-184	aae-miR-184	aae-miR-184
**2**	aae-miR-275-3p	aae-miR-275-3p	aae-miR-317	aae-miR-2940-3p
**3**	aae-miR-317	aae-miR-2940-3p	aae-miR-2940-3p	aae-miR-275-3p
**4**	aae-miR-2940-3p	aae-miR-317	aae-miR-275-3p	aae-miR-317
**5**	aae-miR-276-3p	aae-miR-2940-5p	aae-miR-305-5p	aae-miR-2940-5p
**6**	aae-miR-92b-3p	aae-miR-276-3p	aae-miR-2940-5p	aae-miR-283
**7**	aae-miR-2940-5p	aae-miR-92b-3p	aae-miR-283	aae-miR-276-3p
**8**	aae-miR-281-5p	aae-miR-283	aae-miR-281-5p	aae-miR-281-5p
**9**	aae-miR-283	aae-miR-305-5p	aae-miR-12-5p	*aae-miR-14*
**10**	**aae-miR-989**	aae-miR-281-5p	aae-miR-92b-3p	aae-miR-305-5p
**11**	aae-miR-305-5p	aae-miR-12-5p	aae-miR-998	aae-miR-12-5p
**12**	aae-miR-34-5p	aae-miR-14	aae-miR-2a-3p	aae-miR-92b-3p
**13**	aae-miR-2a-3p	*aae-miR-2765*	**aae-bantam-5p**	*aae-miR-71*-*3p*
**14**	aae-miR-998	aae-miR-998	**aae-miR-8-3p**	*aae-miR-100*
**15**	aae-miR-14	*aae-bantam-3p*	aae-miR-276-3p	aae-miR-2a-3p
**16**	aae-miR-92a-3p	aae-miR-11-3p	**aae-miR-263a-5p**	*aae-miR-11*-*3p*
**17**	aae-miR-12-5p	aae-miR-2a-3p	**aae-miR-989**	*aae-miR-125*-*5p*
**18**	aae-miR-11-3p	aae-miR-1889-5p	**aae-miR-1889-5p**	*aae-miR-306*-*5p*
**19**	aae-miR-1889-5p	aae-miR-92a-3p	aae-miR-970	aae-miR-970
**20**	**aae-miR-306-5p**	aae-miR-34-5p	**aae-miR-92a-3p**	aae-miR-998

miRNAs are arranged by their abundance from the top to the bottom. miRNAs in italic indicate up-regulated miRNAs in the presence of *Wolbachia*, and miRNAs in bold indicate those found only in non-infected cells.

The read numbers for the other 16 most abundant miRNAs varied greatly in *Wolbachia*-infected mosquito cells, changing the sRNA profile in the nucleus and the cytoplasm (Table 1). In addition, *Wolbachia* induced changes that involve the incorporation and disappearance of specific miRNAs. In the nucleus, miR-989 and miR-306-5p were down-regulated and miR-2765 and Bantam-5p were up-regulated. Changes in the cytoplasm were more pronounced, bantam-5p, miR-8-3p, miR-263a-5p, miR-989, miR-1889-5p and miR-92a-3p were down-regulated and dropped from the list of the most abundant miRNAs, while the other six miRNAs were up-regulated; miR-14, miR-71-3p, miR-100, miR-11-3p, miR-125-5p and miR-306-5p (Table 1). Clearly, our results show that *Wolbachia* has an impact on specific miRNAs and changes the natural miRNA profile of the host cell.

#### Induced or suppressed miRNAs

**Figure 8:**
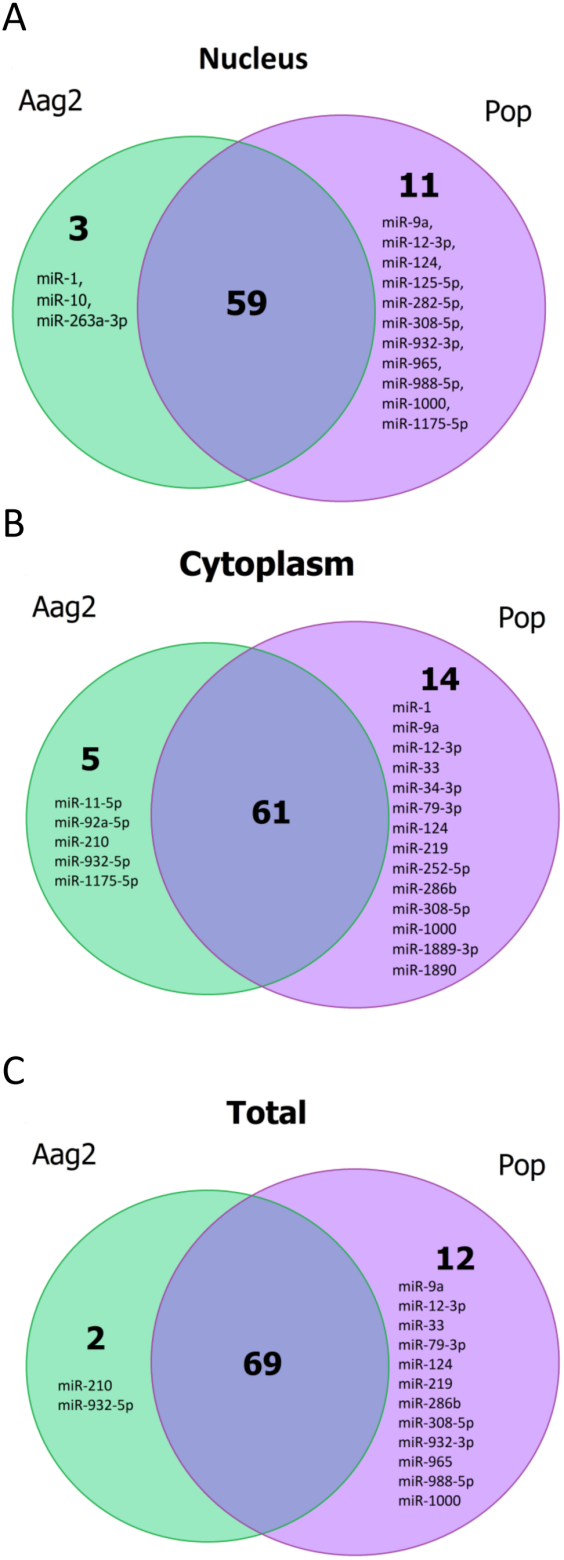
miRNA expression comparison in the nucleus (A), the cytoplasm (B) and the nucleus and the cytoplasm combined (C) between Aag2 and Pop cells.

We also examined the data to find out if the presence of *Wolbachia* affects other than those abundant miRNAs. It was found that those miRNAs with relatively lower number of reads were impacted in a greater manner in the presence of the bacterium. Deep sequencing data revealed that in the nucleus of mosquito cells infected with *Wolbachia*, the expression of miR-1, miR-10 and miR263a-3p was suppressed and 11 other miRNAs were induced; miR-9a, miR-12-3p, miR-124, miR-125-5p, miR-282-5p, miR-308-5p, miR-932-3p, miR-965, miR-988-5p, miR-1000 and miR-1175-5p (Figure 8A). In the cytoplasm, miR-11-5p, miR-92a-5p, miR-210, miR-932-5p and miR-1175-5p were suppressed and 14 miRNAs were induced; miR-1, miR-9a, miR-12-3p, miR-33, miR-34-3p, miR-79-3p, miR-124, miR-219, miR-252-5p, miR-286b, miR308-5p, miR-1000, miR-1889-3p and miR-1890 (Figure 8B). If we consider miRNA reads of the nucleus and the cytoplasm together, we identified a total of 83 miRNAs from which miR-210 and miR-932-5p were suppressed by the presence of *Wolbachia* and 12 were induced; miR-9a, miR-12-3p, miR-33, miR-79-3p, miR-124, miR-219, miR-286b, miR-308-5p, miR-932-3p, miR-965, miR-988-5p and miR-1000 (Figure 8C). Sixty-nine miRNAs were expressed in cells regardless of the presence or the absence of *Wolbachia*. In honeybees, miR-210 has been associated with learning and memory processes such as mushroom bodies and antennal lobe [36], [37]. Also, the miRNA in old foragers, as compared to nurse bees, is up-regulated, therefore it has been suggested that the miRNA participates in the behavioural transition from nursing to foraging [38]. miR-932-5p has been found to be a strong regulator of Hedgehog (Hh) proteins, a highly conserved secreted signalling protein that regulates the growth and patterning of many organs in *Drosophila* and vertebrates. This protein controls cell fates and growth in a concentration-dependent manner [39]. miR-124 is involved in reproduction in *Drosophila* and it is required to ensure fidelity of gender-appropriate pheromone production in males [40]. miR-308 is also implicated in cell proliferation and growth through the tight regulation of the transcription factor *Myc*, ensuring a proper growth of larvae and oogenesis in *Drosophila* [41].

#### Most regulated miRNAs

There was another set of miRNAs that were expressed, but suffered high fluctuations in their copy numbers in response to *Wolbachia*. These are listed and separated according to their cellular compartmentalization in Table 2. As it appears in the list, the highly changed miRNAs in response to *Wolbachia* were miR-125-5p, miR-100, miR-14, miR-276-3p, miR-8-3p, miR-283, miR-317, bantam-5p and miR-263a-5p (Table 1 and Table 2). There were other low expressed miRNAs that were also heavily affected by *Wolbachia*, such as miR-278-3p (Table 2). Hussain et al. (2011) screened 491 insect miRNAs in Tet and Pop mosquitoes using miRNA microarrays, 105 of which were *Ae. aegypti*-specific. They found the abundance of 35 miRNAs (13 from *Ae. aegypti*) was changed. Deep sequencing results from this study are in agreement with those found in mosquitoes and similar results were found for those miRNA changing levels, *i.e*. miR-2941, miR-970, miR-989 and miR-210; however, other miRNAs such as miR-2940 or miR-309 showed differences probably due to the nature of the cell line type that we used for our deep sequencing. miR-2940 was not detected in Tet mosquitoes by Northern hybridization, but it was induced in *Wolbachia* infected mosquitoes [10]. In cells, miR-2940 was present in both Aag2 and Pop cell lines, however, it was up-regulated in the presence of *Wolbachia* (similar to what happens in mosquitoes). It is worth mentioning that miR-2940 is also induced after blood feeding in uninfected mosquitoes (unpublished data). In this study, using more sensitive technique we identified other new miRNAs expressed at lower levels that seem to be highly regulated by *Wolbachia* infection and could be involved in the maintenance of infection.

**Table 2:** Most differentially expressed miRNAs in the nucleus and the cytoplasm of Aag2 cells *versus Wolbachia* infected cells (Pop).

Nucleus					Cytoplasm				
miRNA	Aag2	Pop	Fold	Log2 FoldChange	miRNA	Aag2	Pop	Fold	Log2 FoldChange
**miR-2946**	56.84	602.34	10.60	**3.41**	**miR-125-5p**	19.08	2007.64	105.22	**6.72**
**miR-125-5p**	15.05	147.15	9.78	**3.29**	**miR-100**	81.41	2550.82	31.33	**4.97**
**miR-100**	36.78	295.49	8.03	**3.01**	**miR-71-3p**	225.14	2724.54	12.10	**3.60**
**miR-9c-5p**	23.40	142.96	6.11	**2.61**	**miR-2946**	21.62	246.83	11.41	**3.51**
**let-7**	25.08	130.40	5.20	**2.38**	**let-7**	185.71	1666.48	8.97	**3.17**
**miR-2941**	25.08	126.21	5.03	**2.33**	**miR-14**	597.83	4448.40	7.44	**2.90**
**miR-2765**	315.96	1315.95	4.16	**2.06**	**miR-285**	15.26	88.83	5.82	**2.54**
**miR-277-3p**	28.42	75.37	2.65	**1.41**	**miR-996**	25.44	108.48	4.26	**2.09**
**miR-7**	23.40	57.42	2.45	**1.29**	**miR-281-3p**	26.71	101.40	3.80	**1.92**
**bantam-3p**	511.55	1168.20	2.28	**1.19**	**miR-2941**	125.93	406.40	3.23	**1.69**
**miR-9c-3p**	50.15	101.69	2.03	**1.02**	**miR-9c-5p**	34.34	110.05	3.20	**1.68**
**miR-34-5p**	1721.87	814.69	−2.11	**−1.08**	**miR-276-3p**	2297.20	7077.83	3.08	**1.62**
**miR-281-5p**	4236.14	1875.22	−2.26	**−1.18**	**miR-988-3p**	315.45	643.80	2.04	**1.03**
**miR-263a-5p**	616.87	218.33	−2.83	**−1.50**	**miR-2945-3p**	99.21	48.74	−2.04	**−1.03**
**miR-989**	2495.88	801.53	−3.11	**−1.64**	**miR-278-5p**	80.13	36.16	−2.22	**−1.15**
**miR-34-3p**	35.11	10.17	−3.45	**−1.79**	**miR-278-3p**	716.13	316.79	−2.26	**−1.18**
					**miR-2b**	558.40	246.04	−2.27	**−1.18**
					**miR-8-3p**	2992.97	1256.15	−2.38	**−1.25**
					**miR-283**	20239.77	8318.25	−2.43	**−1.28**
					**miR-998**	4909.85	1774.17	−2.77	**−1.47**
					**miR-190**	290.01	103.76	−2.79	**−1.48**
					**miR-8-5p**	432.47	150.14	−2.88	**−1.53**
					**miR-317**	86651.18	27885.26	−3.11	**−1.64**
					**miR-1175-3p**	48.34	14.94	−3.24	**−1.69**
					**bantam-5p**	3027.32	738.13	−4.10	**−2.04**
					**miR-263a-5p**	2243.78	492.08	−4.56	**−2.19**
					**miR-305-5p**	26004.39	4216.51	−6.17	**−2.62**
					**miR-263a-3p**	87.77	12.58	−6.98	**−2.80**
					**miR-1174**	118.29	15.72	−7.52	**−2.91**

### miRNAs Involved in Host-pathogen Interaction in Mosquitoes

There have been two studies using mosquitoes that examined changes in the levels of specific miRNAs after being challenged with a pathogen. In *Anopheles gambiae*, four miRNAs miR-34, miR-1174, miR-1175, and miR-989 changed their expression levels during *Plasmodium* infection [42]. In *Culex quinquefasciatus*, two miRNAs were detected to change expression levels during the infection with the West Nile Virus (WNV) flavivirus, miR-989 and miR-92 [29]. Notably, miR-989 was affected in both infections with different parasites and different mosquito species as hosts. In our experiments using *Ae. aegypti* and *Wolbachia*, miR-989 was also highly impacted after infection. miR-989 was among the highly expressed miRNAs and was down-regulated in the nucleus and the cytoplasm in *Wolbachia*-infected cells (Table 1). In the nucleus, it was among the most regulated miRNAs with a reduction of 3 times of its levels compared to uninfected cells (Table 2). In *C*. *quinquefasciatus* infected with WNV, miR-989 was down-regulated 2.8 fold, and in *A. gambiae* it was up-regulated 4 times in the midgut and down-regulated to almost half in the rest of the body [29], [42]. The role of this miRNA in host-pathogen interactions is clear from these independent studies (regardless of the nature of the pathogen); however, its targets in mosquitoes are not known yet. The expression of miR-989 was found to be restricted to the ovaries in *Anopheles stephensi* and *Ae. aegypti* [43] and to the midgut in females of *A. gambiae* [42]. Its role in development has also been established, but not in immunity (reviewed in [1], [2]).

The other three miRNAs that changed levels after *Plasmodium* infection in *A. gambiae* also changed abundance in Pop cells. miR-1174, miR-1175 and miR-34 were down-regulated 0.59 to 0.62 times in *Plasmodium* infected mosquitoes [42]. In our system, we found that miR-1174 and miR-1175 were restricted to the cytoplasm of uninfected cells while in *Wolbachia*-infected cells miR-1175 was found only in the nucleus (Figure 8A and B), and miR-1174 was down-regulated 7.5 times to very low levels in the cytoplasm (Table 2). In fact, miR-1174 was the most down-regulated miRNA. In uninfected Aag2 cells, these miRNAs were expressed at relatively low levels and in *A. gambiae* it was only found in the midgut [42]. The targets of miR-1174 and miR-1175 are unknown and therefore further experimental data is needed to clarify their role in host-pathogen interaction.

The levels of miR-92 were altered in *C. quinquefasciatus* mosquitoes infected with DENV, suggesting that its targets may participate in mediating flavivirus infection of the mosquito host [29]. We found miR-92a-3p and miR-92b-3p among the most abundant miRNAs expressed in the nucleus and in the cytoplasm, but miR-92a-5p and miR-92b-5p were detected at very low levels (less than 30 copies) in Aag2 cells (data not shown). Li et al. (2009) reported miR-92a-5p and miR-92b-5p in the embryos and the midgut of *Ae. aegypti* females that were blood fed, linking this miRNA also with embryonic development. miR-92a-3p and miR-92b-3p were down-regulated in *Wolbachia*-infected cells, while miR-92 was up-regulated in *C. quinquefasciatus* infected with DENV [44].

### *Wolbachia* Increases the Modifications of Mature miRNAs

miRNAs are usually reported in the database as single sequences but each miRNA is composed of a sequence heterogeneity that may arise from imprecise precursors cropping or dicing, terminal trimming or the addition of non-template nucleotides [3]. This is a new concept that aroused in the last few years with progressive studies unraveling the mechanisms underlying these modifications [45], [46].

**Figure 9:**
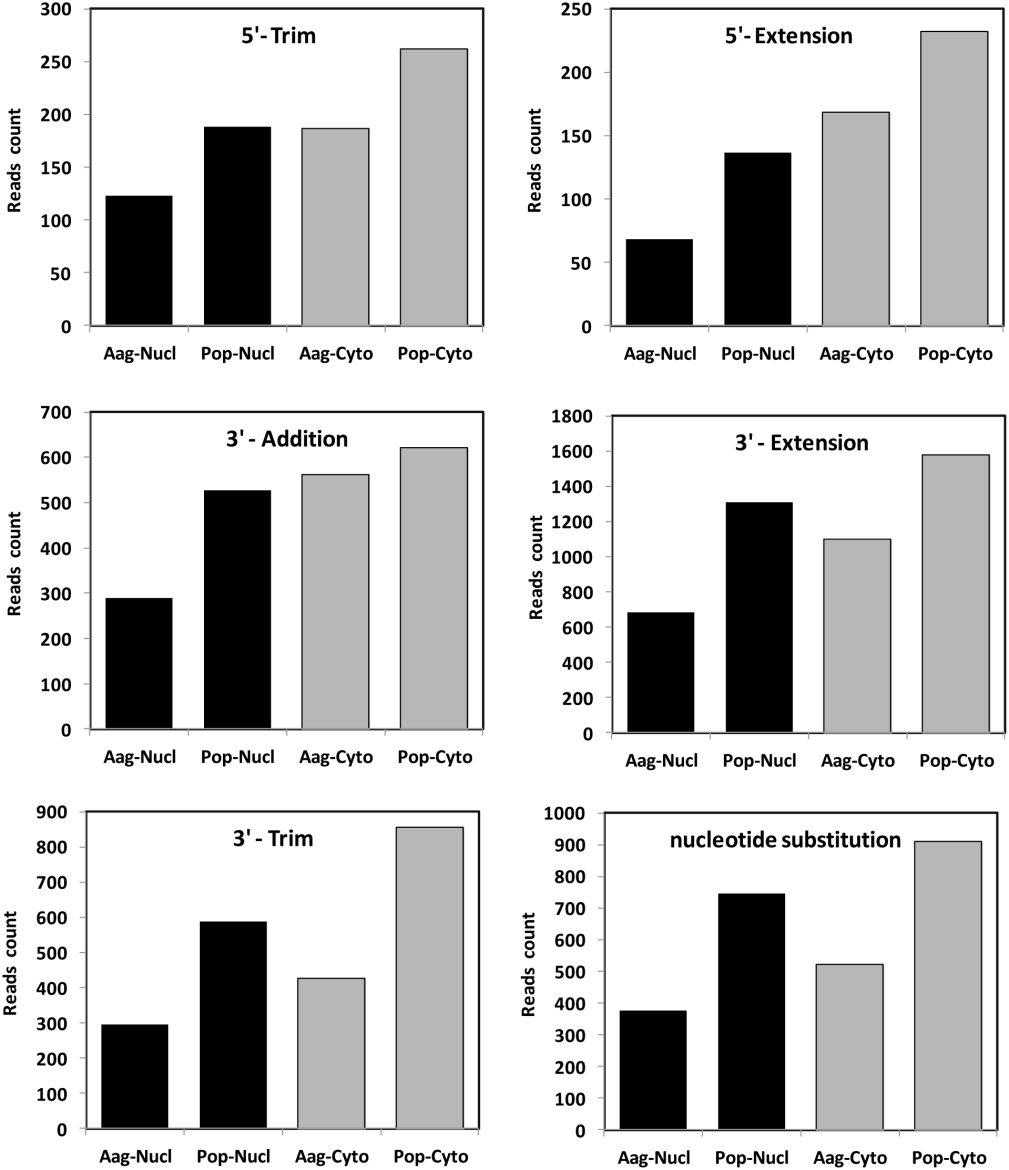
IsomiR analysis of detected miRNAs in four different libraries of *Ae. aegypti* cells in the presence and absence of *Wolbachia.* “5′ trimmed (5′-Trim): The read aligns to a position in the hairpin after the reference mature sequence, but the last base of the read and the mature sequence map to the same position (they end at the same position). 3**′ trimmed (3′-Trim):** the read is shorter than the reference mature sequence, but it starts at the same position in the hairpin. **Nucleotide Substitutions:** Sequence variation between the reference mature sequence and the read. Only those reads are considered that have the same length as the mature sequence, and that start at the same position within the hairpin sequence. **Nucleotide Addition in 3′ (3′-Addition):** The read is at least two nucleotides longer than the reference mature sequence, and the nucleotides do not align with the hairpin sequence**. Nucleotide extension in 3′ (3′-Extension):** The read is at least two nucleotides longer than the reference mature sequence, but the nucleotides align with the hairpin sequence. **Nucleotide extension in 5′ (5′-extension):** The read aligns to a position in the hairpin before the reference mature sequence, but the last base of the read and the mature sequence map to the same position (they end at the same position). For nucleotide addition and extension, the read starts at the same position in the hairpin as the reference mature sequence.

To find out if an external factor, such as a parasite challenge, could lead to modifications in mature miRNA, we studied the extension of nucleotides with respect to the mature form at the 5′ and 3′ ends independently. For this, the extended nucleotides must match to the hairpin loops that they come from (5′- or 3′-extension). We also examined the extension of nucleotides at the 3′ end that did not match the hairpin loop (3′-addition). Trimming of nucleotides at the 5′ and 3′ ends were also evaluated (5′- or 3′-trimming), as well as substitutions in the middle of the mature miRNA sequence (nucleotide substitutions). It was found that for any type of isoform, there were always a bigger number of modifications occurring in the isoforms that were located in the cytoplasm than in the nucleus (Figure 9). Interestingly, the presence of *Wolbachia* increased the number of each type of modification that was analyzed; this was consistent for both cellular compartments but the induction in the nucleus was always more pronounced (Figure 9).

The 3′-extension of mature miRNA by at least two nucleotides was the most frequent type of modification found in our analysis. Similar results were not found in other insects and only a miRNA study of the Atlantic halibut fish *Hippoglossus hippoglossus* studied the 3′- extension. The abundance of four isomers of miR-301c was evaluated. The study concluded that isomers with 2 or 3 template nucleotide additions were more abundant in the early developmental stages. The shorter canonical forms of the miRNA were detected only later in the development of the fish. Independently, Liu et al (2011) reported in *Drosophila* that the abundance of isomers of miR-34 with the same 5′ terminus but different 3′ ends changed during the 54 hours of the experiment. Longer isoforms of 24 nt were more abundant in the early hours of the experiment and miRNAs with the canonical size of 21 nt were detected in high amounts after 33–36 hours [47]. All these data together suggest that there may not be an “extension” in the sequence of specific pools of miRNAs, but a trimming of initially longer fragment of small RNA that after its processing will result into the final canonical size of miRNA. Deep sequencing data indicated that *Wolbachia* inhibited this “trimming”, resulting in pools of miRNAs that are longer than their canonical versions (Figure 9; 3′-extension).

The second most abundant modifications in the deep-sequencing data were nucleotide substitution and 3′-Trimming. Nucleotide substitutions usually correspond to deamination of adenosine to inosine by Adenosine Deaminase Acting on RNA (ADAR) [3]. Kim et al (2010) compiled several examples of miRNAs whose processing by Dicer was affected after being edited by ADAR. It was suggested that editing affects the structure of the small RNA precursors and thereby interferes with its processing. There is not much known about the implications of this modification outside of the seed sequence and therefore the biological significance of miRNA editing remains to be established. Occasionally, RNA nucleotide substitutions occur in the seed sequence of miRNA, changing the target specificity. In the brain of mammals where ADAR is abundant, miR-376 cluster miRNAs are frequently edited in the middle of the 5′ seed region. This area is critical for the hybridization of the miRNA to its targets and it was shown that edited miR-376 silences a different set of genes [48]. The other cause of modification was miRNAs with the same 5′ end but different 3′ ends (3′-Trimming) requiring the 3′-to-5′ exoribonuclease Nibbler. More than a quarter of all *Drosophila* miRNAs undergo 3′ end trimming after being loaded into AGO1. Our deep sequencing data suggests that this may be the case in mosquitoes too, and as highlighted above *Wolbachia*-infected Pop cells showed almost double the number of reads trimmed compared with that of Aag2 cells. Trimming occurs after the removal of the miRNA-3p strand from pre-RISC complex and it was suggested to be the final step in RISC assembly, enhancing target messenger RNA repression [47], [49].

The addition of nucleotides at the 3′ end that do not match to the genome was another form of modification in the mature miRNA sequence. Similar results have been reported for *D. melanogaster* [45] and also in mammalian miRNAs [50], [51], suggesting that a similar mechanism may be acting in insects and mammals. Pre-miRNA and mature miRNA can be modified at their 3′ ends by uridylation and adenylation [52]. In *D. melanogaster*, there is a 2 to 2.5 fold greater modification in 3p species than in 5p species. Furthermore, miRNAs coming from introns (mirtrons) are more frequently modified at the 3′ end than canonical miRNAs [45], [46]. Recent studies implicated the terminal nucleotidyl transferases (TNTases) in this type of 3′-additions, through the uridylation of the 3′ ends. All TNTases are members of the DNA polymerase-β superfamily, which includes the poly(A) polymerase. The biological consequences of the modification are not clear, but several examples compiled by Ameres and Zamore (2013) showed an active role of this modification in the function of the miRNA and the genes they regulated [3], [5].

Modifications at the 5′ end (5′-trimming and 5′-extesion) occurred less frequently. Mehrabadi et al studied the natural modifications of the 5′ end in *Spodoptera frugiperda* and compared them with those at the 3′ end [33]. Similar to our results, the study found that the 5′ end is more conserved with lower number and types of modification than at the 3′ end; however, the study did not analyze the effect of viral infection on the miRNA modifications. The higher conservation of the 5′ end is probably due to the fact that this area contains the seed region, a 6 to 8 nucleotide domain essential for AGO binding and recognition of the target mRNA. Therefore, any modification in this region would have a direct implication in the miRNA-target recognition. It has been suggested that a single nucleotide shift could alter its target repertoire [3]. In addition, the 5′ end of miRNAs is further constrained given that AGO loading typically selects for miRNAs with distinct 5′ nucleotides [3], [5]. To the best of our knowledge, our study represent the first report that points to the effect of *Wolbachia*, or any other pathogen, on the structure of mature miRNAs.

### miRNA Trafficking between the Nucleus and the Cytoplasm

In the sections above, and especially where the miRNA profiles of nuclear and cytoplasmic fractions were compared, different compositions of miRNAs in both cellular compartments were identified. This included a number of miRNAs that were found exclusively in one compartment or the other, with some being present only in the nucleus or enriched in this compartment. These results suggest that there may be an active transport mechanism to shuffle miRNAs between these cellular compartments. Other studies in mammals made similar observations. In human cells, miR-29b is predominantly localized to the nucleus [53]. Another study reported that a substantial fraction of all human miRNAs are present in the nucleus of neural stem cells; they studied the expression of 373 miRNAs and reported that different miRNAs were at different levels in the nucleus and the cytoplasm [54].

In insects, Hussain et al (2013) showed that AGO1 and AGO2 are normally found in the nucleus and the cytoplasm of *D. melanogaster* flies and *Ae. aegypti* mosquito cells [55]. The results showed that in *Wolbachia*-infected cells the translocation of AGO1 into the nucleus was blocked in both species. However, AGO2 migration into the nucleus was not affected in the presence of *Wolbachia* and therefore it was still present in both cellular compartments. Based on these findings, it was hypothesized that AGO2 might be responsible for carrying miRNAs into the nucleus when *Wolbachia* is present. Previous work using *Drosophila* concluded that there is a sorting of miRNA and siRNA loading onto AGO1 and AGO2, respectively; however there are some guide miRNAs and many miRNA-3p strands that were found loaded onto AGO2 as well. *Drosophila* small RNAs are sorted between AGO1 and AGO2 according to their duplex structure and the identity of their first nucleotide [56]. Under this scenario, it is possible to explain the different miRNA profiles that were found between the nucleus and the cytoplasm of Pop versus Aag2 cells, and how some specific miRNAs are selected and actively transported into the nucleus. A good example is miR-276-3p, which was found at 10 times higher levels in the nucleus than in the cytoplasm. For this miRNA and others, AGO1 and AGO2 may have an active role in the trafficking of specific miRNAs between these two cellular compartments and according to Hussain et al (2103) this trafficking is affected by *Wolbachia* [55]. Several other examples also support this hypothesis. For instance, based on our deep sequencing data, miR-1175 was identified only in the cytoplasm of uninfected cells, but it was found only in the nucleus when *Wolbachia* was present (Figure 3A, B). Opposite to that, miR-1 was found in the nucleus in uninfected cells, but in *Wolbachia*-infected cells we could only find it in the cytoplasm (Figure 3A, B). Hussain et al. (2013) showed that in cells infected with *Wolbachia*, translocation of AGO1 into the nucleus is controlled by miR-981, which down-regulates importin β-4 that in turn is involved in AGO1 translocation to the nucleus [55]. It seems possible that miR-1 is one of those miRNAs that is loaded onto AGO1 in the cytoplasm and transported to the nucleus mediated by importin β-4; blocking the distribution of AGO1 to the nucleus by *Wolbachia* explains why this miRNA was not detected in the nucleus of infected cells.

These examples support the concept that there is an active mechanism in insects that transport miRNAs between the cytoplasm and the nucleus and *vice versa*. Translocation of specific miRNAs may be related to specific functions carried out by specific miRNAs in particular cellular compartments.

### *Wolbachia* Modifies the Levels of Specific piRNAs

**Figure 10:**
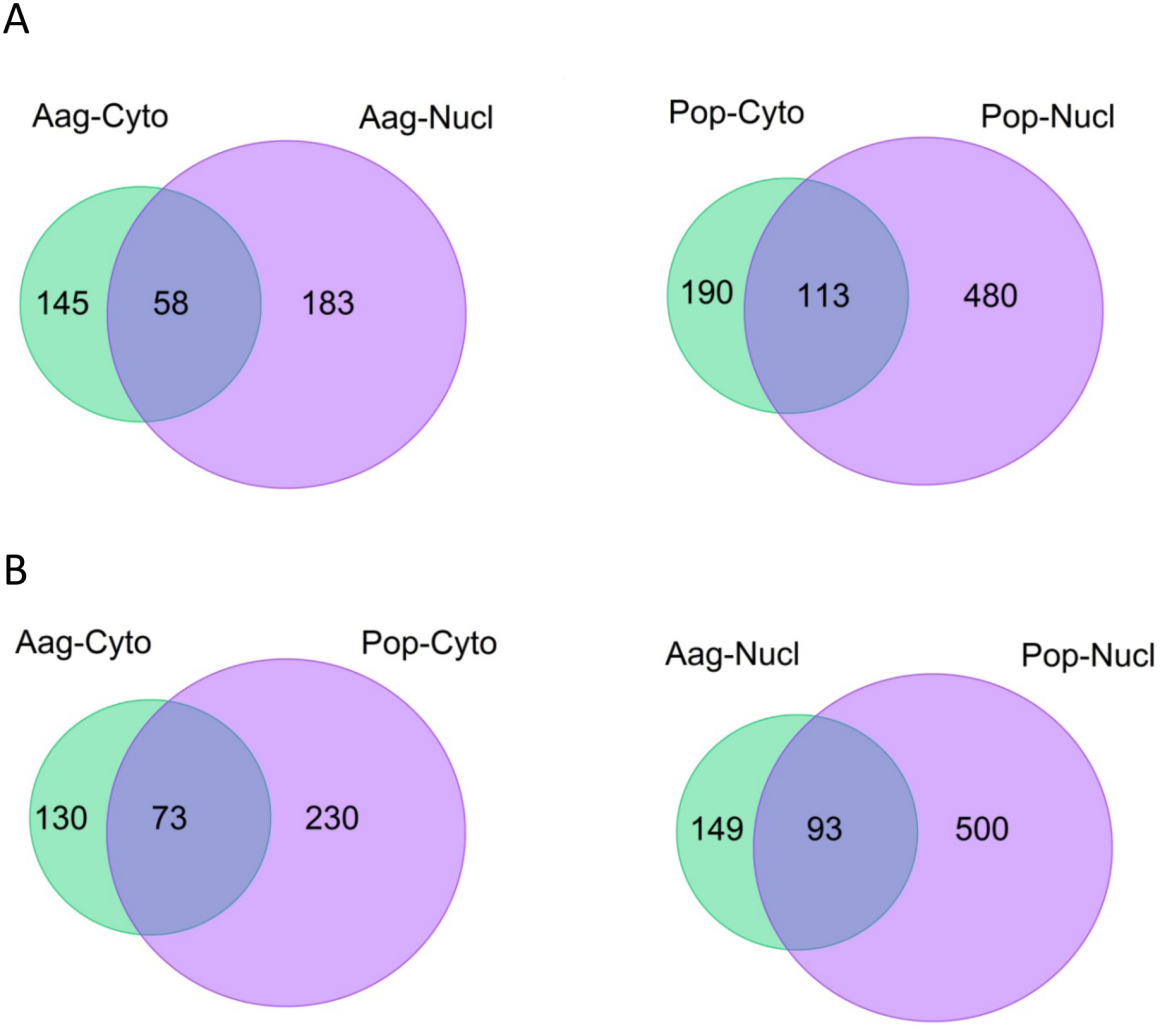
piRNA expression comparison in the nucleus and the cytoplasm of Aag2 and Pop cells. (A) Comparison of cytoplasmic versus nuclear fractions in Aag2 and Pop cells. (B) Comparison of Aag2 versus Pop cells in the cytoplasmic and nuclear fractions.

The presence of piRNA, their cellular compartmentalization and the possibility that they are affected by *Wolbachia* was assessed using our deep sequencing dataset of Aag2 and Pop cells. piRNAs are associated with PIWI proteins, Aubergine, AGO3 and the nuclear Piwi. They are most abundant in the germline of many animal species and piRISC protects the integrity of the genome from transposable elements by silencing them [22]. As noted above, some piRNAs were present in the nucleus and in the cytoplasm of Aag2 and Pop cells, but piRNAs were more abundant in the cytoplasm than miRNAs (Figure 5). Pools of piRNA in the nucleus and the cytoplasm were different and only a portion of them was found in both compartments (Figure 10A). Interestingly and similar to the findings with miRNAs, there was an overall up-regulation of piRNAs in *Wolbachia*-infected cells compared with uninfected Aag2 cells (Figures 5 and 10B). Table 3 and Table 4 summarize the changes in the piRNA levels in the nucleus and the cytoplasm of Aag2 cells compared to Pop cells. It is remarkable that most piRNAs were up-regulated in the nucleus; only piR-197269 was completely suppressed in *Wolbachia*-infected cells and no reads were detected in any of the two cellular compartments examined. In the nucleus, there were 13 piRNAs that were up-regulated while only 6 were up-regulated in the cytoplasm.

**Table 3:** Differentially expressed piRNAs in the nucleus of Aag2 cells versus *Wolbachia* infected cells (Pop).

piRNA*	Normalized expressionvalue in Aag2 cells	Normalized expressionvalue in Pop cells	Log2 FoldChange
PIR5650**	0	12.22396229	∞
PIR59291**	0	8.149308193	∞
PIR8200**	0	2.394752846	∞
PIR54642	0	2.251782527	∞
PIR249348**	0	1.215247713	∞
PIR8813**	0	0.750594176	∞
PIR48167	0	0.714851596	∞
PIR35224	0	0.679109016	∞
PIR37716	0	0.643366436	∞
PIR44728	0	0.536138697	∞
PIR260343	0	0.464653537	∞
PIR86373	0	0.393168378	∞
PIR214814	0	0.357425798	∞
PIR210808	0.042309408	1.322475452	4.966118624
PIR239381	0.042309408	0.536138697	3.663555854
PIR35218	0.042309408	0.500396117	3.564020181
PIR54535	0.042309408	0.357425798	3.078593354
PIR7660	0.084618817	0.571881277	2.756665259
PIR72436	0.761569349	4.503565054	2.564020181
PIR59286	0.126928225	0.428910958	1.756665259
PIR75737	0.507712899	1.429703192	1.493630853
PIR65192**	0.719259941	2.001584469	1.47655734
PIR2224	8.800356922	23.8760433	1.439929833
PIR32635**	0.423094083	1.036534814	1.292718159
PIR31042	0.338475266	0.822079335	1.280227215
PIR224625	0.634641124	1.393960612	1.135176882
PIR22527	0.169237633	0.357425798	1.078593354
PIR202518**	0.761569349	0.357425798	−1.091331648
PIR236686**	1.396210473	0.357425798	−1.965800766
PIR61400	0.423094083	0.107227739	−1.980300335
PIR60123	0.423094083	0.07148516	−2.565262836
PIR197269**	1.057735207	0	−∞

*piRNA name in RNAdb v2.0.

**These piRNAs differentially expressed in both cellular fragments between Aag2 and Pop cells.

**Table 4:** Differentially expressed piRNAs in the cytoplasm of Aag2 cells versus *Wolbachia* -infected cells (Pop).

piRNA*	Normalized expressionvalue in Aag2 cells	Normalized expressionvalue in Pop cells	log 2 FoldChange
PIR8200**	0	10.93765991	∞
PIR59291**	0	10.04478972	∞
PIR5650**	0	2.678610591	∞
PIR8813**	0	2.008957943	∞
PIR249348**	0	1.785740394	∞
PIR31228	0	1.67413162	∞
PIR32635**	0.199082548	1.339305296	2.7500462
PIR19840	0.99541274	3.906307112	1.972438621
PIR42038	0.398165096	1.339305296	1.7500462
PIR82944	0.796330192	2.678610591	1.7500462
PIR58103	1.99082548	0.781261422	−1.349489474
PIR69440	2.588073124	0.892870197	−1.535356019
PIR238999	2.189908028	0.669652648	−1.709385419
PIR236686**	18.51467696	4.910786084	−1.914643494
PIR65192**	140.950444	35.37998156	−1.994182821
PIR202518**	6.370641536	1.45091407	−2.134476583
PIR51286	3.185320768	0.446435099	−2.834916301
PIR60603	3.782568412	0.223217549	−4.082843814
PIR37488	1.99082548	0.111608775	−4.156844396
PIR197269**	6.171558988	0	−∞

*piRNA name in RNAdb v2.0.

**These piRNAs were differentially expressed in both cellular fragments between Aag2 and Pop cells.

It has been reported recently that virus-derived piRNAs (vpiRNAs) are produced after viral infection in mosquito and mosquito cells [24]–[27]. In these studies, several thousands of reads with 25–29 nt that mapped to viral genomes were sequenced. Their biosynthesis is still not completely understood but they seem to have the classical ping-pong signature present in piRNA of eukaryotes, suggesting that vpiRNAs may be processed by similar mechanisms as canonical piRNAs. When piRNA pathway proteins were depleted, the replication of Semliki Forest virus increased by several folds [26]. These results suggested that non-canonical piRNA pathway may be present in mosquitoes and may be acting redundantly to the siRNA pathway to target virus replication. These are the first reports that study the involvement of piRNA in host-pathogen interaction. However, none of these studies evaluated the effect of the pathogen on the endogenous levels of the mosquito piRNAs. In our study, changes in specific pools of mosquito piRNAs were detected in the presence of the pathogen. Our results using *Wolbachia* suggests that host piRNAs may also be involved in this interaction. Further functional experiments will elucidate the role of host piRNAs after *Wolbachia* infection.

## Conclusions

This study found an overall increase for small RNAs between 18 and 28 nucleotides in cells infected with the endosymbiontic bacterium *Wolbachia* strain *w*MelPop-CLA, and also an increase in the mRNA levels of the argonaute proteins AGO1–3. *Wolbachia* changed the abundance of specific sets of miRNAs in the nucleus and the cytoplasm of mosquito cells and consequently altered the natural miRNA profile of Aag2 cells. We have identified for the first time pools of piRNAs that were affected by the presence of *Wolbachia*, but the implications of these changes need further studies. The different miRNA profiles found between the nucleus and the cytoplasm suggests that there is an active transport of miRNAs between cellular compartments; furthermore this shuttling of miRNAs between compartments was affected by the presence of *Wolbachia*, changing also the abundance of specific miRNAs in the nucleus and the cytoplasm. Analysis of the structure of the mosquito miRNAs showed different types of modifications naturally occurring in the insect cells, but interestingly *Wolbachia* increased the frequency of these modifications; the implications and reasons of these manipulations are unknown and need further characterization. Overall, this work contributes to the understanding of the complicated host-endosymbiont interactions and provides new data to better understand how *Wolbachia* manipulates the host RNAi/miRNA/piRNA machinery in order to facilitate its persistent replication in mosquito cells.

## Supporting Information

Figure S1 **1% Denaturing agarose gel of RNA in the nucleus and cytoplasmic fractions from Aag2 and Pop cells.** 5S specific probe to the nuclear fraction was used in Northern blot to confirm integrity and purity of the nuclear RNA.
(TIF)

Table S1 **A complete list of all miRNAs identified in the four libraries with their count numbers.**
(XLSX)
