# Supplementary figures and images for: Wolbachia Infection Modifies the Profile, Shuttling and Structure of MicroRNAs in a Mosquito Cell Line
Source: PLoS One. 2014 Apr 23;9(4):e96107. doi: 10.1371/journal.pone.0096107 (PMC3997519; doi:10.1371/journal.pone.0096107)

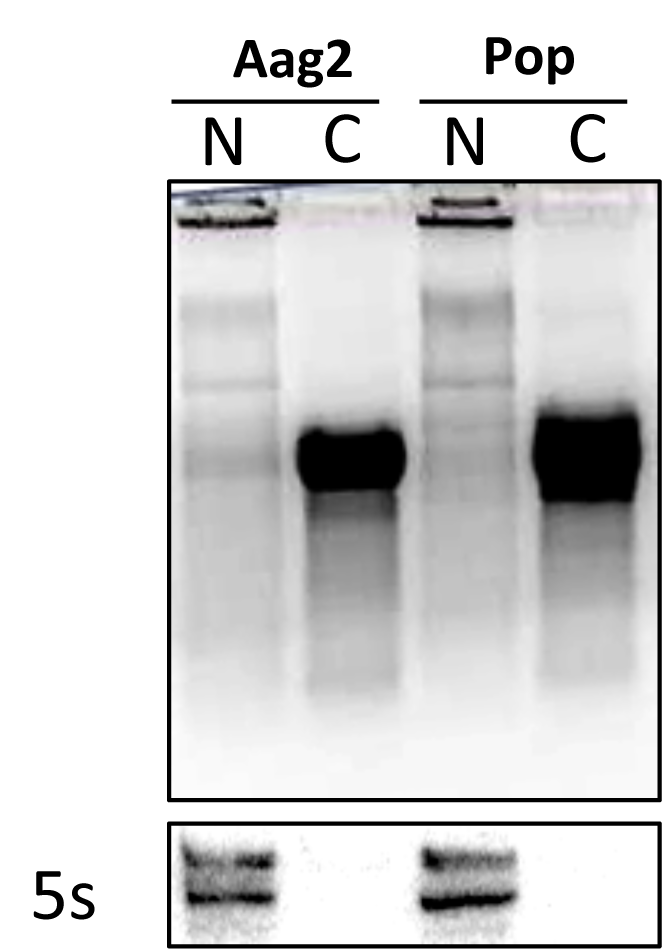

Supplement: Figure S1 — 1% Denaturing agarose gel of RNA in the nucleus and cytoplasmic fractions from Aag2 and Pop cells. 5S specific probe to the nuclear fraction was used in Northern blot to confirm integrity and purity of the nuclear RNA. (TIF) [file pone.0096107.s001.tif]
